# Dopaminergic nigrostriatal vulnerability in Parkinson’s Disease with diabetes: evidence from severity-matched cohorts

**DOI:** 10.1007/s00259-026-08011-0

**Published:** 2026-06-20

**Authors:** Alice Galli, Cinzia Zatti, Alessandro Lupini, Silvia Paola Caminiti, Andrea Rizzardi, Silvia Lucchini, Francesco Bertagna, Barbara Paghera, Tiago Fleming Outeiro, Daniela Perani, Alessandro Padovani, Andrea Pilotto

**Affiliations:** 1 Neurology Unit, Department of Clinical and Experimental Sciences, University of Brescia, Brescia, 25123 Italy; 2 Department of Continuity of Care and Frailty, Neurology Unit, ASST Spedali Civili of Brescia, Brescia, 25123 Italy; 3 Laboratory of Digital Neurology and Biosensors, University of Brescia, Brescia, 25123 Italy; 4 Department of Brain and Behavioral Sciences, University of Pavia, Pavia, Italy; 5 Nuclear Medicine Unit, University of Brescia, Brescia, 25123 Italy; 6 Department of Experimental Neurodegeneration, University Medical Center Goettingen, Goettingen, Germany; 7 Max Planck Institute for Experimental Medicine, Goettingen, Germany; 8 Institute of Neuroscience, The Medical School, Newcastle University, Framlington Place, Newcastle Upon Tyne, NE2 4HH UK; 9 Vita Salute San-Raffaele University, Milan, 20123 Italy; 10 Brain Health Centre, University of Brescia, Brescia, 25123 Italy; 11 Department of Neurobiology, Care Sciences and Society (NVS), Center for Alzheimer Research, Division of Clinical Geriatrics, Karolinska Institutet, Stockholm, Sweden

**Keywords:** Parkinson’s disease, Diabetes mellitus, Dopamine transporter imaging, Nigrostriatal pathway

## Abstract

**Purpose:**

Diabetes Mellitus (DM) has been recognized as a potential risk factor and disease-modifier in Parkinson’s Disease (PD), being associated with worse motor and cognitive outcomes, and altered susceptibility of neural pathways. This study investigated the impact of DM on nigrostriatal dopaminergic vulnerability independently of disease severity in drug-naïve PD patients.

**Methods:**

This study analyzed two independent cohorts of PD patients (multi-center PPMI *n* = 174, single-center UniBS *n* = 95). Patients with and without DM were first compared and then matched for age, sex, and clinical severity. All patients underwent baseline ^123^I-FP-CIT imaging to quantify dopamine transporter binding. Dopaminergic binding, neural reserve index and molecular connectivity patterns were compared between severity-matched groups.

**Results:**

Patients with DM were older, predominantly male, and exhibited worse non-motor and cognitive symptoms. After severity matching, PD-DM exhibited more preserved nigrostriatal dopamine uptake compared to PD-n. PD-DM also showed fewer nigrostriatal dopaminergic connectivity alterations (10% vs. 21%) and reduced neural reserve index in the left putamen and – only in the single-center cohort- whole striatum.

**Conclusion:**

In drug-naïve PD patients, comorbid diabetes is associated with comparable clinical severity despite milder dopaminergic loss. This suggests an increased dopamine system vulnerability linked to DM, reducing the efficiency and compensatory mechanisms of nigrostriatal dopaminergic networks in PD.

**Supplementary Information:**

The online version contains supplementary material available at https://doi.org/10.1007/s00259-026-08011-0.

## Introduction

Parkinson’s Disease (PD) is a complex and heterogeneous condition in terms of clinical presentation and patterns of progression. Several pre-morbid factors have been advanced to explain this heterogeneity, namely life-style factors [1], genetics [2], and cardio-vascular factors [3, 4]. Recently, Diabetes Mellitus (DM) has been increasingly recognized as both a potential risk factor and disease-modifier in PD and related alpha-synucleinopathies. The association between DM, worse motor symptoms, and poorer treatment responses in PD patients was first described in an observational study [5] and confirmed even in de-novo PD patients [6, 7]. Additionally, DM has been identified as a powerful predictor of cognitive symptoms and dementia in PD [6, 8, 9]. Shared mechanisms underlying this association have been extensively studied. Firstly, experimental models have demonstrated that prolonged hyperglycemia can be a risk factor for PD pathogenesis and progression, potentially leading to nigrostriatal synaptic dysfunction, increased alpha-synuclein aggregation, and consequent neuroinflammation [10, 11]. Additionally, recent studies have identified possible shared genetic susceptibility mechanisms between DM and PD, putting individuals at risk for both diseases. For instance, the *park7* gene related to PD encodes the DJ-1 protein, which is reduced in pancreatic cells of patients with DM [12] and may have deglycase activity [13]. Also, mitochondrial dysfunction and oxidative stress can contribute to onset and progression of both conditions [14]. Taken together, these findings indicate that DM can increase susceptibility to changes in neural pathways implicated in PD, thus reducing brain’s resilience against neurodegeneration. The concept of brain resilience, originally advanced for brain changes related to normal age or Alzheimer’s Disease, has been recently expanded to PD encompassing the concepts of cognitive and motor reserve [15, 16]. Motor reserve represents an individual’s capacity to maintain motor functionality despite a significant degree of neuropathology in the underlying motor circuitry. The neural basis of motor reserve is the restructuring of both functional and structural brain networks driven by their use throughout life [17].

Here, we hypothesized that DM may affect nigrostriatal pathways, modulating the efficiency and compensatory capacity of nigrostriatal dopaminergic network. We therefore conducted an in vivo study using ^123^I-FP-CIT SPECT imaging in two independent cohorts, applying a severity-matching procedure to isolate the role of DM on nigrostriatal system independently from symptoms severity. To achieve this aim, we used complementary analytical strategies, namely the evaluation of regional differences in ^123^I-FP-CIT binding in nigrostriatal pathway and the assessment of their molecular connectivity alterations in clinically matched PD patients with and without concomitant DM.

## Methods

### Participants

The study evaluated the relationship between DM and dopaminergic function in two independent cohorts of drug naïve patients: (i) Parkinson’s Progression Markers Initiative (PPMI) database, a multicenter, longitudinal study (www.ppmi-info.org/data); (ii) Internal validation data derived from the ongoing single-center observational Digital Neurodegenerative Assessment (DNA) study conducted at the Neurology Unit of the University of Brescia (UniBS), Italy.

## Multicenter PPMI cohort

Drug-naïve PD patients were selected from the PPMI dataset according to the following inclusion criteria: (i) no prior use of dopaminergic medications (i.e., dopamine agonist or Levodopa); (ii) absence of other concomitant neurological disorders; (iii) absence of cognitive decline or dementia; (iv) no history of cerebrovascular events; (v) no concomitant anti-depressant medications; (vi) availability of baseline clinical assessment; (vii) availability of medical history reporting vascular risk factors. Further up-to-date details on general inclusion criteria, protocols and manuals are available online at www.ppmi-info.org/study-design.

An age and sex-matched control group (CG) of healthy subjects was selected as normative dataset for brain dopaminergic analysis (*n* = 56, age = 60.48 ± 10.08, sex (M) = 62%). According to the enrollment criteria, healthy subjects had no current or past clinically significant neurological disorders, no first-degree relatives with Parkinson’s disease, and normal ^123^I-FP-CIT SPECT imaging on visual inspection.

### Clinical assessment

All participants underwent a comprehensive evaluation, including a physical examination and assessment of motor, non-motor, and cognitive symptoms. To evaluate the severity of motor symptoms, the MDS Unified Parkinson’s Disease Rating Scale- Part III (MDS-UPDRS-III) was considered. Non-motor symptoms were evaluated using the MDS-UPDRS-part I, and the Rapid Eye Movement (REM) Sleep Behavior Disorder Questionnaire (RBDSQ). Global cognition was assessed using the Montreal Cognitive Assessment (MoCA). Cardiovascular risk factors were collected from medical history or home therapy, namely, hypertension, hypercholesterolemia, smoking status, and Body Mass Index (BMI). Type 2 DM status was identified based on clinical history and confirmed by fasting plasma glucose measurements ≥ 126 mg/dL obtained at screening and baseline visits, in line with standard diagnostic criteria.

### ^123^I-FP-CIT SPECT procedures

All individuals underwent ^123^I-FP-CIT SPECT scan at baseline to assess dopaminergic alterations. All scans were acquired using Siemens or General Electric SPECT tomographs, approximately 3–4 h after the administration of ^123^I-FP-CIT tracer (see “SPECT technical operations manual”). Brain SPECT scans pre-processing was conducted using Statistical Parametric Mapping (SPM12), running on MATLAB R2022b according to a previously validated pipeline [18]. First, the origin coordinates of the DAT-SPECT scans were manually set to the anterior commissure. Then, each image was spatially normalized to a high resolution ^18^F-DOPA template (http://www.nitrc.org/projects/spmtemplates) using the old-normalize function. Parametric binding potentials were generated for each subject and voxel-wise using the Image Calculator (ImCalc) function, considering the superior lateral occipital cortex as the reference background region. Spatial smoothing with an 8 mm FWHM Gaussian kernel was applied to parametric images only for subsequent voxel-wise analyses to minimize the partial volume effect.

### Single-center UniBS cohort

Drug-naïve PD patients were retrospectively included from the DNA cohort according to specific inclusion criteria: (i) formal diagnosis of PD [19]; (ii) no prior use of dopaminergic medications (i.e., dopamine agonist or Levodopa), (iii) absence of cognitive decline or dementia; (iv) absence of other concomitant neurological or psychiatric disorders; (v) no history of cerebrovascular events; (vi) availability of medical history reporting vascular risk factors.

An age and sex-matched CG (*n* = 30, age = 72.47 ± 4.51, sex (M) = 50%) for brain dopaminergic analysis was selected from an internal normative dataset of subjects with a confirmed clinical diagnosis of isolated action or rest tremor syndromes over a 4-year follow-up period and normal ^123^I-FP-CIT imaging on visual inspection and semi-quantitative analysis. This normative set has been partially described in previous works [18, 20].

## Clinical assessment

Participants underwent comprehensive clinical evaluations. The same clinical scales as the PPMI cohort were selected for consistency. Cardiovascular risk factors were collected from medical history or home therapy. Specifically, type 2 DM diagnosis was established based on documented medical history and/or ongoing antidiabetic treatment (e.g., metformin or other glucose-lowering medications).

### ^123^I-FP-CIT SPECT procedures

Brain SPECT acquisition was performed 3 h after tracer administration using the Discovery 630, General Electric, Milwaukee, WI. Data were reconstructed by filtered back-projection, with Butterworth 3-dimensional (3D) post-filter (order 10.0; cut-off 0.50 cycle/cm) and corrected for attenuation (Chang’s method coefficient 0.13 cm-1). The same pre-processing pipeline was applied to ^123^I-FP-CIT SPECT scans as for the PPMI cohort for consistency, considering lateral occipital region as background reference region to obtain parametric images.

## Statistical Analysis

First, PD patients with and without DM (PD-DM, PD-n) were compared for demographic and clinical variables using ANOVA test – adjusting for age and sex - or chi-squared test for categorical variables. The normality assumption was assessed using the Shapiro-Wilk test and all considered variables met the assumption (*p* > 0.05).

*Matching Procedure-* to test the effect of DM independently of clinical severity, PD without DM (PD-n) were matched 1:1 to PD-DM based on sample size, age, sex, and clinical symptoms severity, separately for the two cohorts. Of note, motor symptoms (MDS-UPDRS-III), non-motor symptoms (MDS-UPDRS-I) and cognitive symptoms (MoCA) were retained in the matching procedure performed using the MatchIt library in R (nearest neighbour approach). The same matching procedure was also applied to select an age and sex-matched CG for each cohort for subsequent brain dopaminergic analyses.

Significance threshold was set at *p* < 0.05. Statistical analyses were performed using R (version 4.3.1). All univariate statistical analyses were conducted separately within each cohort to avoid biases related to data acquisition protocols.

### Brain dopaminergic analysis

#### Univariate specific binding ratio (SBR) comparisons

*ROI-based comparisons -* Parametric SBRs were extracted following a previously validated pipeline [18] from basal ganglia (i.e., bilateral putamen and caudate). SBR mean values were extracted from each designated region of interest (ROI) applying a map of dopamine peaks in CG as conjunction mask using REX toolbox (https://www.nitrc.org/projects/rex/). ROIs were selected from the Automated Anatomical Labelling (AAL) atlas [21]; anterior and posterior putamen were derived from the Harvard Oxford subcortical Atlas available in FSL (https://fsl.fmrib.ox.ac.uk/fsl/fslwiki/Atlases). Individual z-scores using CG as reference population were calculated, with lower z-scores indicating reduced SBRs values.

SBR in the posterior putamen was considered to calculate asymmetry index (AI%) adopting the formula by Walker et al. [22]. Group differences in mean SBR and AI% were assessed using ANOVA with CG as reference. Additionally, mean putamen SBR was compared between patients with and without concomitant hypertension or hypercholesterolemia using separate ANOVA models (see Supplementary Materials).

*Voxel-wise comparisons-* PD-DM and PD-n were directly compared using a complementary voxel-wise independent-sample t-test model in SPM12. Given the supportive and exploratory purpose of the voxel-wise analysis, the significance threshold was set at *p* < 0.01 (uncorrected) combined with a cluster-extent criterion of 100 voxels to avoid spurious voxels. This voxel-wise analysis was intended to provide spatial support to the ROI-based results.

#### Neural reserve index calculation

Neural reserve operational measures were calculated by modelling the relationship between motor symptoms and nigrostriatal dopaminergic uptake to derive an individual’s expected level of dopaminergic alteration based on motor symptoms severity. Dopaminergic uptake was quantified within (i) the whole striatum, (ii) regions that resulted significant in the prior univariate SBR comparisons. The mean SBR was extracted from the corresponding ROIs selected from the AAL atlas. Of note, the whole striatum SBR was computed as the average SBR across bilateral caudate, putamen, and pallidum.

The following linear regression models were fitted separately for each considered cohort:

$\:{Mean\:SBR}_{ROI}\:\sim\:{UPDRS}_{III}+Age+AI\%$. Standardized residuals were derived from each of these regression models and multiplied by -1 to derive W scores, obtaining an operational measure of neural reserve according to the following formula:

$$\:W=-1*\frac{Observed\:{SBR}_{ROI}-Predicted\:{SBR}_{ROI}}{SD}$$ [23].

The distribution of W scores was compared between PD-DM and PD-n by means of Welch’s test (significant threshold *p* < 0.05). Lower W scores were considered as a proxy of lower neural reserve, meaning that a relatively lower level of brain damage is tolerated at a given level of motor symptoms.

#### Multivariate molecular connectivity analyses

Molecular connectivity was estimated following the principle that neurotransmitter release is correlated among territories receiving dopaminergic projections from the same afferents. 

*Nodes selection –* Specific cortical and subcortical ROIs known to be rich in dopamine transporter [24] were selected a priori from the AAL atlas and were considered as nodes of the nigrostriatal dopaminergic circuit [25]. Of note, only nodes with a mean SBR significantly greater than zero in the CG were retained for subsequent analyses. Mean SBR in each ROI considered as node was compared between groups using ANOVA (see Supplementary Materials).

Assessment of molecular connectivity between nodes of nigrostriatal dopaminergic pathway was performed via Pearson’s correlation analysis [18, 26]. ROI-to-ROI Pearson’s correlation matrices were estimated using MATLAB’s *corr* function separately for patients and controls in the PPMI and UniBS cohorts. Prior to connectivity (i.e., correlation) estimation, regional SBR values were z-standardized within each cohort to minimize inter-cohort variability related to potential acquisition and reconstruction differences. The estimated Pearson’s correlation coefficients were considered as edges (i.e., strength of the connectivity between nodes). Fisher z-transformation was applied to correlation coefficients, which were then compared between clinical groups using standard error-based z-tests. The significant threshold was set at *p* < 0.05 [27], with and without FDR correction for multiple comparisons. The obtained z-scores can be negative or positive, indicating significantly decreased or increased connectivity, respectively [28]. Thus, a symmetric and weighted adjacency matrix was obtained for each comparison. The percentage of connectivity alterations was calculated for each comparison by considering the number of significant z-scores in the adjacency matrix normalized to the total number of possible pairwise connections (i.e., number of edges). 

*Graph Theory Analyses -* To assess and describe molecular network topology, specific local (nodal) metrics were analyzed using BRAPH software (version 2.0.0.b1) [29]. In detail, the network topology was examined converting each undirected adjacency matrix into a binary matrix, to apply a connectivity density (i.e., sparsity) threshold (S), retaining the top S% connections (binary undirected density method, BUD). To avoid biases associated with using a single threshold and to ensure the robustness of graph metrics, a range of thresholds was examined (15% < S < 30% in steps of 5%) [30]. At each of these thresholds, the following local nodal metrics were extracted:

1. *Nodal degree (fundamental metric)*: the total number of edges connected to a node, reflecting its connectivity strength;
2. *Path length (fundamental metric)*: the average distance from a given node to all other nodes;
3. *Local efficiency (functional metric)*: the measure of the efficiency of the subgraph defined as the average of the shortest inverse distances among nodes within the local subgraph (all neighboring nodes of a given node and all edges among them).

For all metrics, non-parametric tests with *n* = 1000 permutations were carried out to assess differences between groups, which were considered significant for a two-tailed test of the null hypothesis at *p* < 0.05.

## Results

### Baseline clinical assessment

**Fig. 1:**
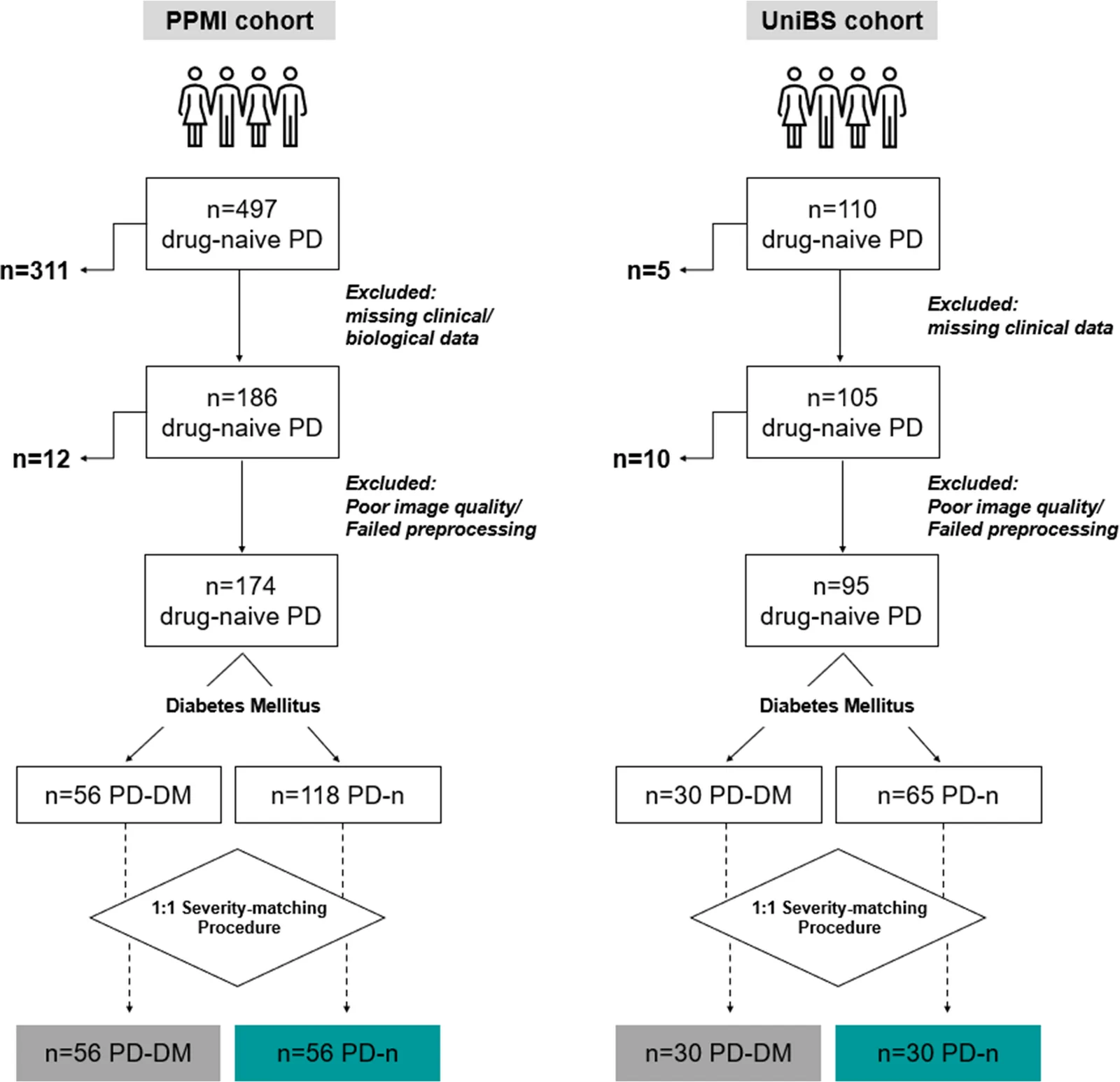
Flowchart representing patients’ enrollment and the final sample of the two independent cohorts. On the left side is reported the number of patients excluded in each step as reported. (left) PPMI cohort: out of 497 PD participants included, 186 drug-naïve PD individuals met the selection criteria for the presence of vascular risk factors and glucose levels at baseline. Twelve out of the 186 patients showed artifacts in dopaminergic imaging and were subsequently excluded from further analyses. The total sample included 174 patients, namely 56 with and 118 PD patients without DM. (right) DNA cohort: Out of the 110 PD participants included, 5 patients were excluded for the absence of clinical data (i.e., vascular factors at baseline). 10 PD patients were further excluded due to artifacts in dopaminergic imaging, resulting in a final sample of 95 patients, namely 30 with and 65 PD patients without DM. Abbreviations: PD, Parkinson’s Disease; UniBS, University of Brescia; PPMI, Parkinson’s Progression Markers Initiative cohort; DM, Diabetes Mellitus

In the multicenter PPMI cohort, the final sample included 174 PD patients, namely 56 with and 118 without DM (see the flowchart of patients’ inclusion- Fig. 1). At baseline, PD-DM were found to be older (*p* = 0.009), have a higher percentage of males (83%, *p* = 0.002), and exhibit worse non-motor (MDS-UPDRS-I, *p* = 0.011) and cognitive symptoms (MoCA, *p* = 0.035) than those with PD-n. Moreover, PD-DM showed more frequent hypertension, hypercholesterolemia, and higher BMI than PD-n. (see Supplementary Table 1, Supplementary Table 2).

The single-center UniBS cohort included a final eligible sample of 95 patients, namely 30 and 65 PD patients with and without DM, respectively (Fig. 1). At baseline, PD-DM were slightly older (*p* = 0.060), had a higher percentage of males (73%, *p* = 0.035), and exhibited worse cognitive symptoms (MoCA, *p* = 0.039) compared to patients without DM. PD-DM also showed more frequent hypertension, hypercholesterolemia, and higher BMI than PD-n (Supplementary Table 1, Supplementary Table 2).

*Matching Procedure-* For subsequent analyses, a 1:1 matching of PD-n with PD-DM patients was performed separately in each cohort as previously described. As expected, after the matching procedure, PD-DM and PD-n were comparable for age, sex, disease duration, motor and non-motor symptoms (Table 1).

**Table 1:** Demographic and clinical characteristics of the included patients after the matching procedure

	PD-DM	PD-*n*	*p*-value
PPMI cohort (n)	56	56	-
Age (mean ± SD)	64.73 ± 8.61	62.99 ± 10.97	0.513
Sex (F/M)	9/47	7/49	0.589
PD Disease duration (years)	2.16 ± 1.88	2.22 ± 2.24	0.857
MDS-UPDRS-I (mean ± SD)	5.88 ± 4.29	5.05 ± 2.69	0.460
MDS-UPDRS-III (mean ± SD)	22.61 ± 2.14	21.07 ± 8.94	0.512
Hoehn and Yahr stage	1.69 ± 0.54	1.64 ± 0.49	0.652
MoCA (mean ± SD)	26.21 ± 2.91	27.05 ± 2.37	0.150
UniBS cohort (n)	30	30	-
Age (mean ± SD)	69.88 ± 7.27	69.27 ± 5.76	0.722
Sex (F/M)	8/22	11/19	0.405
PD Disease duration (years)	1.52 ± 1.30	0.61 ± 1.05	0.904
MDS-UPDRS-I (mean ± SD)	6.17 ± 4.90	5.31 ± 4.98	0.508
MDS-UPDRS-III (mean ± SD)	12.93 ± 9.21	14.17 ± 7.07	0.563
Hoehn and Yahr stage	1.50 ± 0.71	1.33 ± 0.49	0.359
MoCA (mean ± SD)	25.70 ± 2.84	26.07 ± 1.64	0.543

*PD* Parkinson’s Disease, *DM* Diabetes Mellitus, *MDS-UPDRS* Movement Disorder Society Unified Parkinson’s Disease Rating Scale, *MoCA* Montreal Cognitive Assessment. Disease duration was calculated considering the year of symptoms onset as reference.

### Brain dopaminergic activity

#### Univariate SBR comparisons

**Fig. 2:**
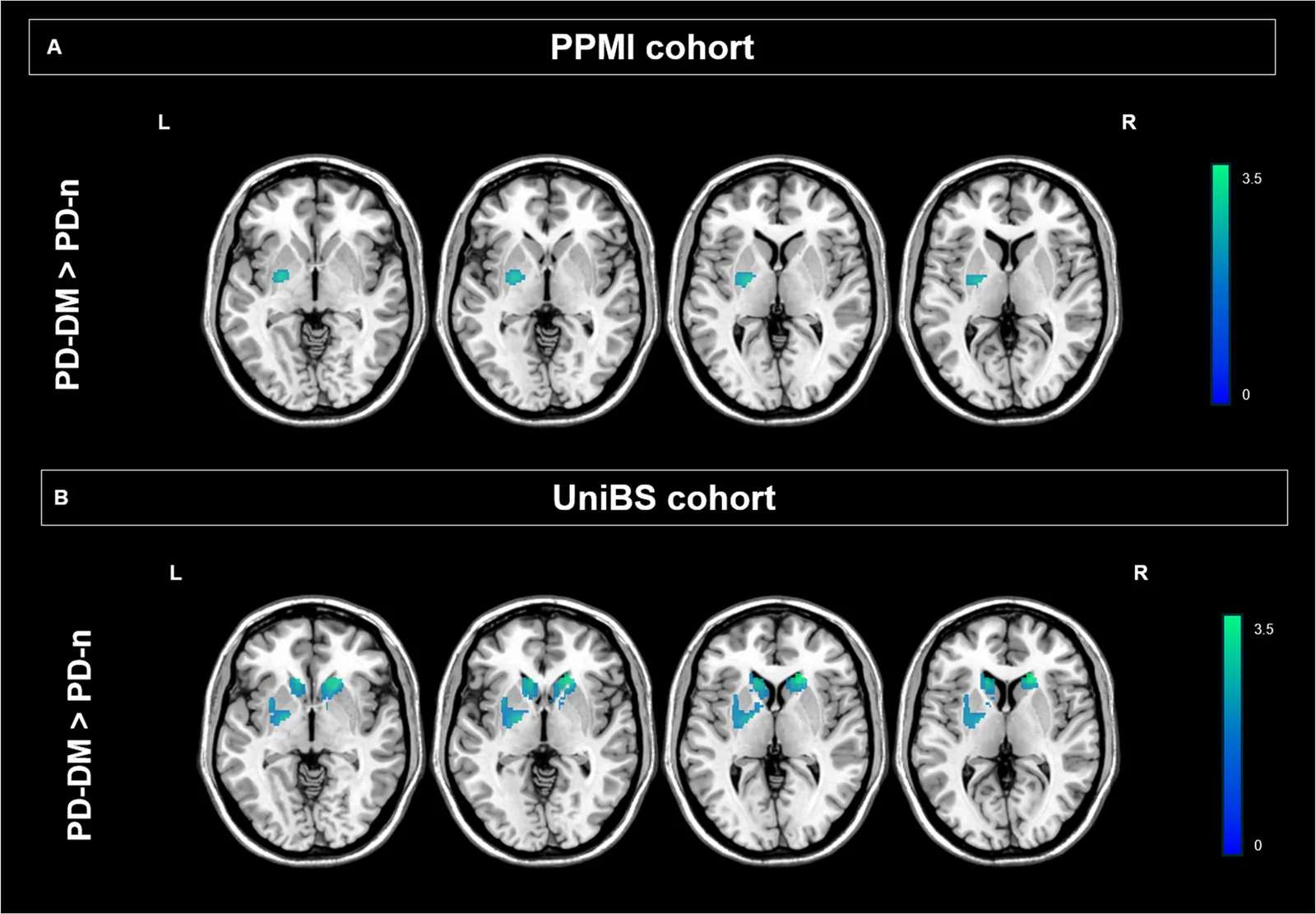
Voxel-wise SPM comparison between PD-n and PD-DM. Significant decreased dopaminergic binding in PD-n as compared to PD-DM in basal ganglia overlaid on a T1-standard template. This voxel-wise analysis was performed to provide spatial support to the region-of-interest findings. Results are represented separately for PPMI (panel **A**) and UniBS (panel **B**) cohorts. Abbreviations: PD, Parkinson’s Disease; DM, Diabetes Mellitus; L, Left; R, Right.

In the multicenter PPMI cohort, both PD-DM and PD-n showed significant dopaminergic reductions within bilateral putamen (*p* < 0.001) and caudate (*p* < 0.001) as compared to CG. The SBR direct comparison showed more preserved mean SBR values in the left putamen (*p* = 0.023) in PD-DM as compared to PD-n with similar cognitive and motor severity (see Table 2). In the voxel-wise supportive analysis, PD-DM exhibited more preserved dopamine uptake in left putamen and pallidum (*p* < 0.01 peak level and *p* < 0.05 cluster level; Supplementary Table 3, Fig. 2).

**Table 2:** Dopaminergic binding in basal ganglia at baseline

	CG	PD-DM	PD-*n*	*p*-value	Post-hoc
PPMI cohort (n)	56	56	56	-	-
Putamen L	0.00 ± 0.00	-2.12 ± 1.36	-2.53 ± 0.92	< 0.001	PD-DM < CG (*p* < 0.001), PD-n < CG (*p* < 0.001), PD-DM > PD-n (*p* = 0.023)
Putamen R	0.00 ± 0.00	-2.07 ± 1.33	-2.24 ± 0.99	< 0.001	PD-DM < CG (*p* < 0.001), PD-n < CG (*p* < 0.001)
Caudate L	0.00 ± 0.00	-1.37 ± 1.22	-1.46 ± 0.86	< 0.001	PD-DM < CG (*p* < 0.001), PD-n < CG (*p* < 0.001)
Caudate R	0.00 ± 0.00	-1.34 ± 1.04	-1.36 ± 0.78	< 0.001	PD-DM < CG (*p* < 0.001), PD-n < CG (*p* < 0.001)
Asymmetry Index (%)	7.97 ± 7.48	16.61 ± 10.27	12.23 ± 13.33	< 0.001	PD-DM > CG (*p* < 0.001)
UniBS cohort (n)	30	30	30	-	
Putamen L	0.00 ± 0.00	-1.24 ± 1.13	-1.81 ± 1.45	< 0.001	PD-DM < CG (*p* < 0.001), PD-n < CG (*p* < 0.001), PD-DM > PD-n (*p* = 0.047)
Putamen R	0.00 ± 0.00	-1.39 ± 1.22	-1.86 ± 1.15	< 0.001	PD-DM < CG (*p* < 0.001), PD-n < CG (*p* < 0.001)
Caudate L	0.00 ± 0.00	-0.11 ± 1.15	-0.63 ± 1.29	0.125	*-*
Caudate R	0.00 ± 0.00	-0.26 ± 1.18	-0.69 ± 1.38	0.075	*-*
Asymmetry Index (%)	5.99 ± 5.27	23.04 ± 12.99	21.44 ± 15.11	< 0.001	PD-DM > CG (*p* < 0.001), PD-n < CG (*p* < 0.001)

Post-hoc comparisons are Bonferroni corrected for multiple comparisons; only significant comparisons are reported in the table. Data are expressed as z-scores computed using the CG as reference group. Abbreviations: *PD* Parkinson’s Disease, *DM* Diabetes Mellitus, *L* Left, *R* Right

In the single-center UniBS cohort, both PD-DM and PD-n exhibited reduced mean SBR in bilateral putamen (*p* < 0.001) than CG. The direct comparison confirmed higher mean SBR values in left putamen in PD-DM patients than PD-n (*p* = 0.047) (see Table 2). The supportive voxel-wise analysis showed higher dopamine uptake in bilateral putamen and caudate in PD-DM vs. PD-n (*p* < 0.01 at peak level and *p* < 0.05 at cluster level; Supplementary Table 3, Fig. 2).

In both the considered cohorts, only DM but not hypertension and hypercholesterolemia status showed a significant effect on mean SBR within bilateral putamen (Supplementary Table 4, Supplementary Table 5).

### Neural reserve index

**Fig. 3:**
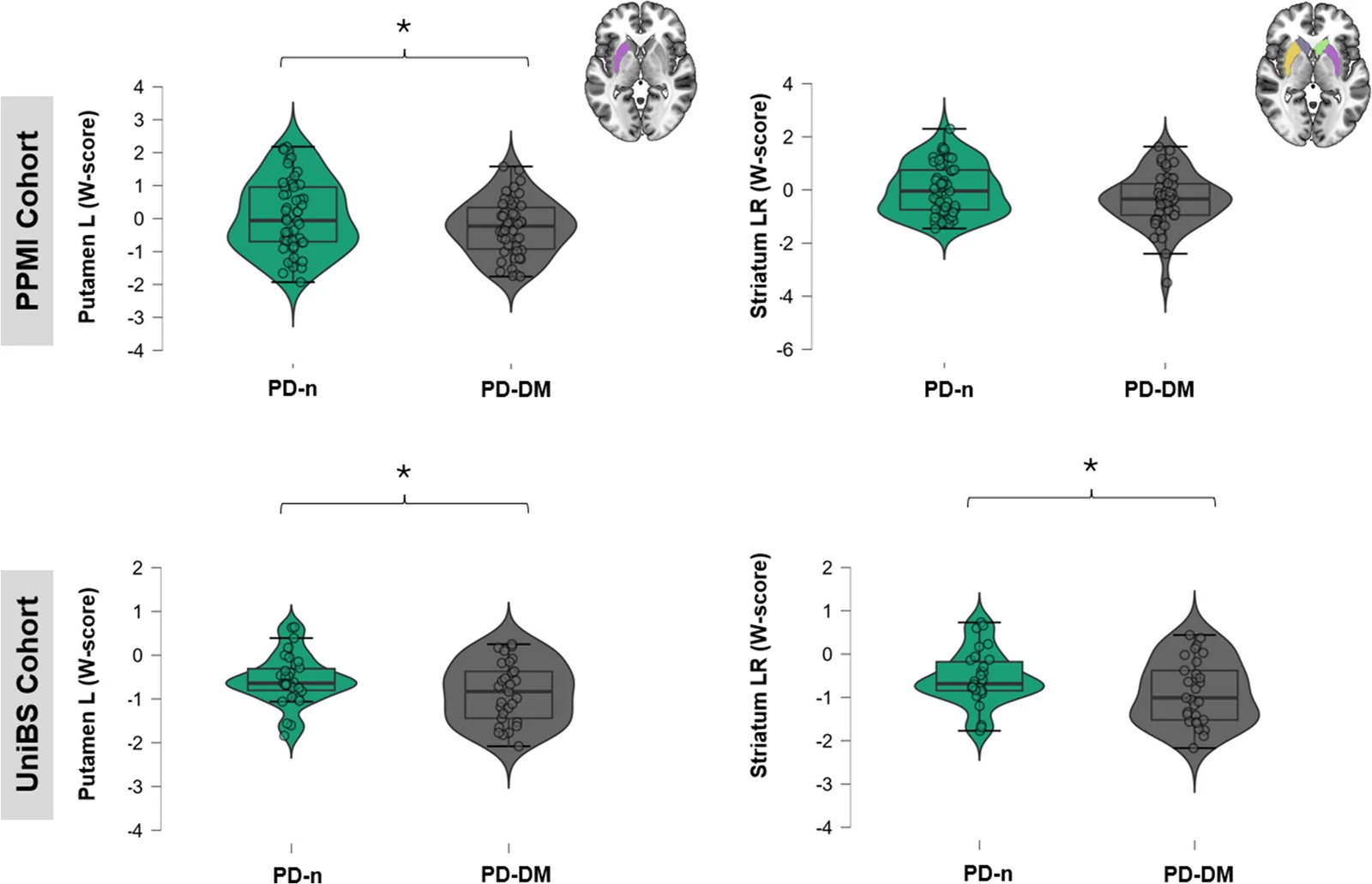
Neural reserve index. Violin plots represent the differences between neural reserve operational measures (i.e., W-scores) for the left putamen and the whole striatum, separately for the two cohorts. Lower W-scores represent lower neural reserve, indicating that a relatively lower level of brain damage is tolerated at a given level of motor symptoms. Abbreviations: PD, Parkinson’s Disease; DM, Diabetes Mellitus; PPMI, Parkinson’s Progression Markers Initiative cohort; UniBS, University of Brescia internal cohort.

The comparison revealed a trend toward lower whole striatum W-scores in PD-DM compared to PD-n (PPMI, *p* = 0.125; UniBS, *p* = 0.031), although this difference reached statistical significance only in the UniBS cohort. For the left putamen, PD-DM showed significantly lower W-scores than PD-n in both cohorts (PPMI, *p* = 0.034; UniBS, *p* = 0.048). Overall, PD-DM tended to show lower W-scores than PD-n, suggesting reduced neural reserve (Fig. 3).

### Multivariate molecular connectivity analysis

Bilateral putamen, caudate, globus pallidus, thalamus, precentral gyrus, and supplementary motor area were considered as nigrostriatal nodes, obtaining *n* = 72 possible unique pairwise connections (i.e., considering the symmetry of the correlation matrix). Lateral frontal cortex showed mean SBR values not significantly different to zero in CG and, therefore, was excluded from further comparisons. Mean ^123^I-FP-CIT SBR for all ROIs considered as nodes are reported in Supplementary Table 6. Cortical motor regions showed a relatively preserved binding in both PD-DM and PD-n vs. CG, whereas striatal regions showed significantly lower ^123^I-FP-CIT SBR in both clinical groups.

#### Specific alterations in PD-DM

Compared to CG, the molecular connectivity analysis demonstrated significant connectivity alterations in the 10% out of all possible connections in PD-DM (*p* < 0.05). Connectivity alterations encompassed only decreased connectivity (i.e., z-scores < 0). Of note, PD-DM exhibited mostly short-distance subcortical alterations between putamen, caudate and pallidum, including contralateral homologous projections. Additional long-range connectivity reductions were observed between the caudate and supplementary motor area. After FDR correction, only short-distance subcortical connectivity losses remained statistically significant.

#### Specific alterations in PD-n

PD-n showed statistically significant altered connections in the 21% of possible connections, as compared to CG. Among these altered connections, the majority (87%) represented decreased connectivity involving short-range subcortical projections among caudate, putamen, and pallidum, as well as long-range connections between caudate and supplementary motor area. PD-n also demonstrated significantly increased connectivity primarily involving projections between globus pallidus and supplementary motor area and intra-precentral gyrus connections. Following FDR correction, decreased connectivity between homologous subcortical regions and increased connectivity between globus pallidus and supplementary motor area remained significant.

#### Direct comparison between PD-DM and PD-n

The direct comparison revealed only relatively lower connections strength in the 8% of nodes in PD-DM as compared to PD-n between precentral gyrus, thalamus, and caudate.

**Fig. 4:**
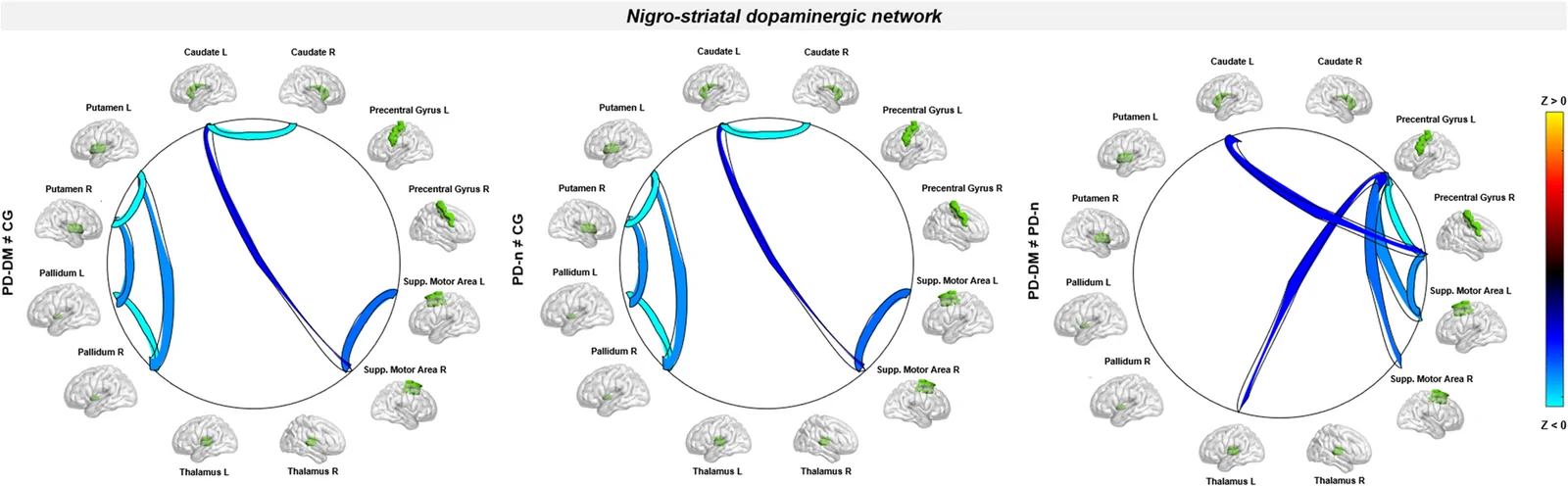
Molecular connectivity analysis results. Network plots representing significant z-matrices comparisons between groups (statistical threshold *p* < 0.05) within nigro-striatal network. Brain renderings represent cortical and subcortical networks’ nodes. Cyan-blue: negative z-scores; Yellow-red: positive z-score. 3D renderings were obtained from BrainNet Viewer toolbox [31]. Abbreviations: PD, Parkinson’s Disease; DM, Diabetes Mellitus; CG, Control Group; L, left; R, right

Multivariate comparisons are depicted in Fig. 4.

**Fig. 5:**
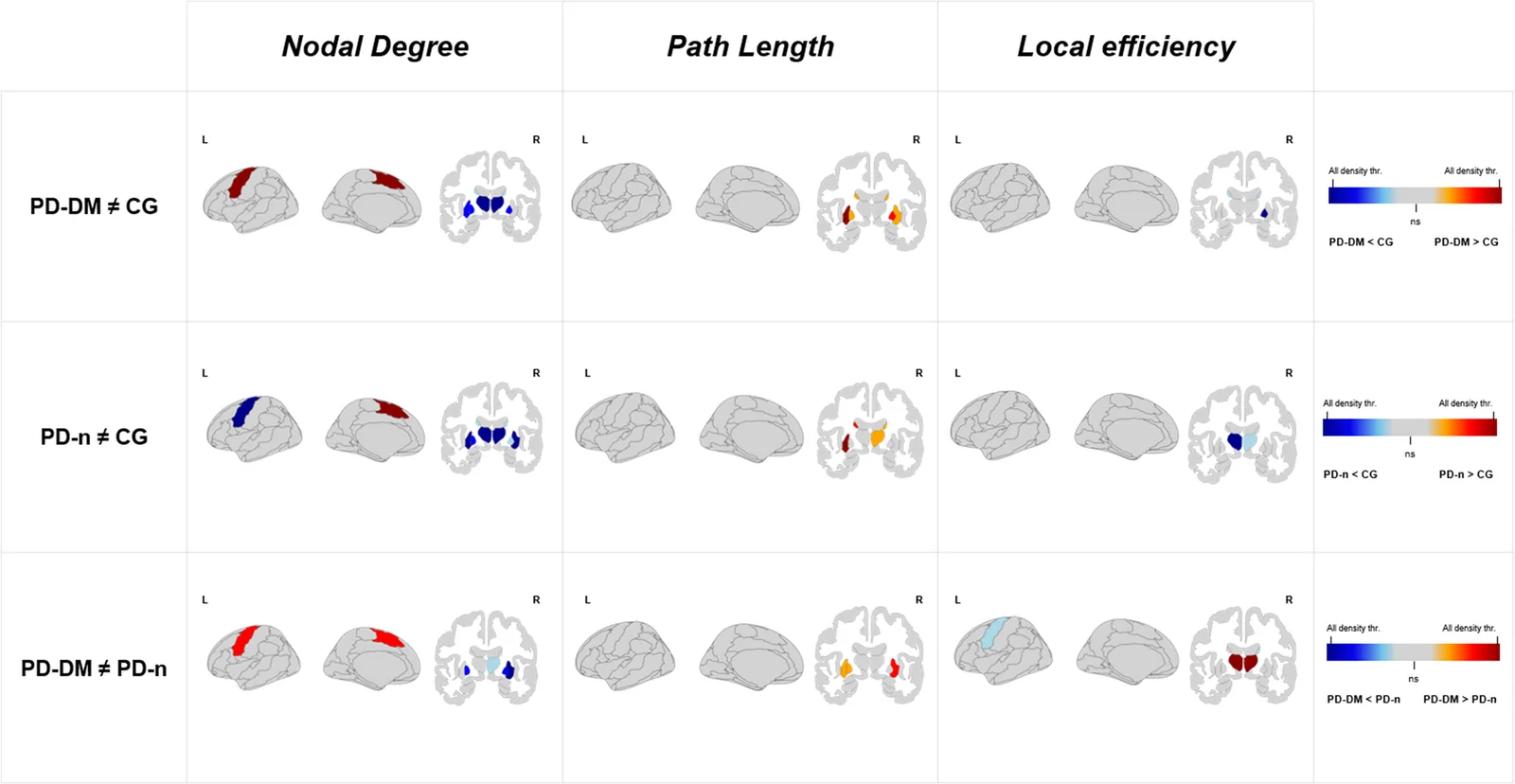
Graph Theory Measures. Brain regions showing significant differences in graph-theoretical measures across multiple network density thresholds. Columns display effects for each extracted nodal metric. Rows correspond to group comparisons: PD-DM vs. CG, PD-n vs. CG, and PD-DM vs. PD-n. Colors indicate direction of the effect (red = increased, blue = decreased) and robustness across the 15–30% density threshold range (dark color = consistently significant in ≥ 3 density thresholds, medium color = significant in 2 density thresholds, light color = significant in 1 density threshold). ggseg-suite packages from the Lifebrain EU project *(*https://www.lifebrain.uio.no/*) *were used for rendering.Abbreviations: PD, Parkinson’s Disease; DM, Diabetes Mellitus; CG, Control Group; L, left; R, right; ns, non-significant; thr., thresholds

*Graph Theory Metrics-* Across the examined density range (15%-30%), both PD-DM and PD-n showed significant alterations in nodal metrics. Reduced *nodal degree* (i.e., reduced number of connections starting from the node) was primarily observed in subcortical nodes (i.e., caudate, globus pallidus, putamen, thalamus) both in PD-DM and PD-n as compared to CG. At the cortical level, PD-n showed decreased *nodal degree* in the precentral gyrus compared to CG, whereas PD-DM exhibited increased *nodal degree* in the same region. These findings were consistent across the density range examined. *Path length* was significantly increased (i.e., lower strength in connections starting from the node) in the bilateral globus pallidus (right: at 30% and 25% density threshold; left: at 30% density threshold), caudate (at 30% density threshold) and putamen (right: at 30% density threshold; left: at all density thresholds) in PD-DM compared to CG. Similarly, *path length* was significantly increased in PD-n vs. CG in bilateral caudate (right: at 30% density threshold; left: at 30% and 25% density threshold), right thalamus (at 30% density threshold) and left putamen (all density thresholds). *Local efficiency* was significantly decreased in the right globus pallidus (all density thresholds) and in bilateral caudate (only at 30% density threshold) in PD-DM vs. CG. Moreover, it is significantly decreased in the bilateral thalamus (right: at 30% density threshold; left: at all density thresholds) (see Fig. 5).

The direct comparison between the two groups of PD patients revealed relatively decreased *nodal degree* in the right putamen (in all densities), bilateral globus pallidus (at 15% and 20% density threshold) and right thalamus (at 30% density threshold) in PD-DM as compared to PD-n. Moreover, the nodal *degree* within the precentral gyrus was relatively lower in PD-n as compared to PD-DM (at 25% and 30% density thresholds). The *path length* was relatively higher in PD-DM vs. PD-n in bilateral putamen (right: at 25% and 30% density thresholds; left: at 30% density threshold) and left pallidum (at 30% density threshold). The *local efficiency* was relatively lower in PD-DM vs. PD-n in right caudate and left precentral gyrus (at 30% density threshold). Finally the *local efficiency* was relatively lower in PD-n vs. PD-DM in bilateral thalamus, consistently across all density thresholds (see Fig. 5).

## Discussion

Diabetes is a known risk factor and modulator of PD, yet the mechanisms underlying this association remain under-investigated. In this study, two independent cohorts of PD patients, with and without concomitant DM, were matched for motor and non-motor symptoms severity. PD-DM exhibited a distinct pattern of nigrostriatal dopaminergic binding and connectivity changes compared to PD-n, providing in-vivo evidence of the potential detrimental effect of DM on nigrostriatal network in PD.

In the present study, PD-DM showed worse non-motor and cognitive symptoms at baseline compared to PD-n, in both the tested cohorts. This finding aligns with previous research linking DM to faster cognitive decline and higher risk for Mild Cognitive Impairment in PD [32], potentially due to mechanisms such as insulin resistance [32], mitochondrial dysfunction [33] or neuroinflammation [34], as demonstrated also for other neurodegenerative conditions [35]. These factors could be potentially influenced also by the large and significant overlap with other comorbidities including cardiovascular diseases, and metabolic syndrome [3, 4].

Growing literature based on animal and in vitro models suggests that DM may induce neurodegeneration by making the nigrostriatal pathway more vulnerable and less resilient [7, 36]. For instance, dysregulation of glucose metabolism can appear years before motor symptoms, suggesting the crucial role of hyperglycemia in facilitating the onset of PD [37]. Notably, previous in vitro and animal studies have described the presence of insulin receptors in the basal ganglia and substantia nigra [33], as well as the key role of insulin in regulation of the dopaminergic transmission [38].

To evaluate the specific effect of diabetes on dopaminergic networks independently from direct (and indirect) relationship with symptoms severity, an accurate matching procedure was applied in both cohorts. Our results revealed in vivo significant differences in dopaminergic nigrostriatal pathway between severity-matched PD-n and PD-DM. First, PD-DM patients showed a higher dopamine binding in basal ganglia, in comparison to PD-n, suggesting that PD-DM need a lower degree of dopaminergic loss to reveal comparable levels of disease severity (assessed/matched using MDS-UPDRS-I, III, and MoCA). This result was further supported by the complementary voxel-wise analysis, demonstrating higher dopamine binding in PD-DM in basal ganglia compared to severity-matched patients in both cohorts. Notably, a previously validated method [23] was applied to obtain an operational measure of interindividual differences in the tolerance of nigrostriatal dopaminergic deficits at comparable levels of motor impairment. Indeed, W-scores were computed to obtain each individual’s neural reserve index calculated as the difference between predicted and observed dopaminergic alterations within the whole striatum and the left putamen (i.e., region obtained from univariate comparisons). PD-DM showed lower W-scores, thus suggesting a lower tolerance for dopaminergic neuron loss at a given level of motor severity. Interestingly, these effects were more pronounced in the left hemisphere, in line with the asymmetrical pattern of dopaminergic degeneration in PD [39, 40], which often shows greater involvement of the left striatum, particularly in early stages of the disease [41, 42]. The consistency between the two cohorts provides robustness to the findings, as they differ for design, recruitment strategies (multi-center vs. single-center) and image settings. Of note, the PPMI cohort included patients with slightly longer disease duration (approximately 2 years vs. 1.5 years in the UniBS cohort), younger age but overall greater motor severity (21.72 ± 10.1 vs. 13.55 ± 8.2 MDS-UPDRS-III for PPMI and UniBS cohorts respectively). Notably, only in the PPMI cohort both PD-DM and PD-n showed significant dopaminergic alterations involving also the caudate nucleus perhaps consistently with the more severe motor symptoms and differences in image acquisition. Despite these differences, several methodological steps were adopted to ensure comparability across cohorts, including the use of a standardized image preprocessing pipeline, cohort-specific analyses, and data normalization procedures, thereby supporting the robustness and generalizability of the findings.

The multivariate dopaminergic connectivity analysis further helped to clarify the relationship between nigrostriatal network nodes beyond regional deficits. Of note, PD-DM showed less prominent molecular connectivity alterations within nigrostriatal pathway (10% of possible connections are altered vs. CG) as respect to PD-n (21%), supporting the univariate analysis findings. Only decreased connectivity characterized the significant alterations in PD-DM vs. CG, mostly involving short-distance subcortical but also long-distance connections. Conversely, PD-n showed increased connectivity in the 13% of altered connections, namely globus pallidus projections to cortical motor regions and connections between them. Interestingly, cortical motor regions showed relatively preserved ^123^I-FP-CIT binding in both PD-DM and PD-n when compared to CG. Given the marked dopaminergic impairment of striatal structures and the relative preservation of cortical motor areas, these connectivity changes are unlikely to reflect primary cortical dopaminergic pathology. Rather, they may represent network-level alterations driven by regions with ^123^I-FP-CIT bindings known to be reduced in PD. This may be consistent with the role of gained connections as an early phase response to counterbalance the neurodegenerative processes [18, 43] described in both Alzheimer’s Disease and Parkinson’s Disease [44]. The advanced hypotheses suggest an ongoing compensatory or maladaptive mechanism due to underlying pathological process.

The direct comparison between PD with and without concomitant DM demonstrated the presence of a relatively more decreased connectivity in PD-DM affecting thalamus and frontal nodes, crucial regions for motor and non-motor symptoms onset. Weaker connections involving the supplementary motor area may contribute to a reduced motor adaptability often reported in patients with PD and concomitant cardiovascular comorbidities. Although it does not survive the FDR correction, there is a tendency toward a more pronounced loss of long-distance connections in PD-DM vs. PD-n, which may suggest worse compensatory mechanisms within nigrostriatal network. Additionally, graph theory metrics were investigated to describe quantitatively the topography of network alterations driven by diabetes mellitus. As expected, some common alterations were found in the comparison of each group of PD patients with controls. Specifically, reduced *nodal degree* in subcortical nodes may indicate a loss of integrative capacity within basal ganglia, while increased *path length* (i.e., lower connections strength) may be consistent with impaired communication within nigrostriatal pathway as compared to CG. Importantly, direct comparisons revealed a more pronounced disruption of network organization in PD-DM compared to PD-n. Relatively reduced *nodal degree* in the putamen and globus pallidus, together with a relatively increased *path length* and reduced *local efficiency* in PD-DM vs. PD-n may indicate a less integrated and less efficient network architecture. Interestingly, the *local efficiency* - a measure of the ability of neighbouring nodes to maintain effective and efficient communication- is reduced in PD-DM vs. PD-n, particularly in crucial regions such as the caudate and precentral gyrus. This finding aligns with the interpretation of impaired local compensatory mechanisms driven by DM. The complex interplay between metabolic dysregulation and neurological health has been also described in a previous study conducted in individuals without PD, showing functional resting-state connectivity abnormalities in fronto-striatal circuits (i.e., reduced connectivity between striatum and motor cortices) in patients with DM [45].

This apparently paradoxical finding - namely comparable clinical severity despite relatively preserved dopaminergic binding - may thus suggest that diabetes influences compensatory and resilience mechanisms rather than solely accelerating dopaminergic degeneration itself. In this context, metabolic dysfunction and insulin resistance may promote striatal oxidative stress, alter dopamine neurotransmission, and increase the chance of nigrostriatal damage [46, 47]. Consequently, clinical manifestations in PD-DM may emerge at relatively lower levels of dopaminergic loss because of impaired adaptive network responses.

Taken together, these findings highlighted the potential role of DM as a modifier of brain vulnerability and resilience in PD, especially within the dopaminergic networks.

These results have several research and clinical implications. First, findings highlight the importance of early screening and management of vascular and metabolic risk factors in the general population, and specifically in subjects at-risk for PD. The increased risk of developing PD in subjects with diabetes prompted its inclusion in the prodromal PD criteria [48–50]. Still, insulin resistance and diabetes screening are often neglected in neurological settings, potentially limiting an important early window of intervention. Patients with both PD and DM are known to experience faster progression of cognitive symptoms, gait disturbances, and postural instability [34]. Studying mechanisms underlying the association between PD and DM may also raise the possibility of new therapeutic targets acting both on metabolic changes and neurodegeneration. Insulin has demonstrated protective effects on dopaminergic neurons in experimental models [49] and Glucagon-like peptide 1 (GLP-1) agonists have reported a significant neuroprotective effect in preclinical studies of PD by reducing neuroinflammation and oxidative stress [51]. The present findings suggest a specific negative role of DM- on nigrostriatal system in PD and potentially support GLP-1 treatments, which should be definitively further explored in early disease phases as target engagement in on-going clinical trials in early PD [52, 53] .

We acknowledge some limitations of the study. First, the absence of correction for partial volume effects might have led to subtle localization biases. However, we used anatomical atlas for ROI segmentation and used non-smoothed images only for ROI-based analyses [18, 54]. Second, we use dopaminergic imaging as proxy of motor reserve in PD but the integration with a clinical scale will be important and has been included in ongoing prospective studies. Moreover, regions included in the W-score analysis were partially selected based on prior univariate SBR results. Although this approach allows a more targeted characterization of clinically relevant regions, it may introduce a data-driven selection bias and limit the full independence of the analysis. Third, detailed diabetes-related variables, including diabetes subtype, disease duration, glycemic control (e.g., HbA1c), diabetes-related complications, and specific antidiabetic treatments, were not systematically available across cohorts and therefore could not be included in the analyses. As a consequence, diabetes mellitus was treated as a binary variable. This is particularly relevant considering the potential neuroprotective effects of some glucose-lowering therapies, such as GLP-1 receptor agonists, which may influence neurodegeneration and dopaminergic imaging findings. In addition, DM was treated as a binary variable in the present analyses. Although this approach allowed us to isolate the effect of diabetes presence on nigrostriatal vulnerability, the neural impact of metabolic dysfunction is likely modulated by factors such as glycemic control, and treatment exposure. In addition, although subjects with major vascular burden or a history of stroke were excluded and key vascular risk factors were considered, the lack of direct neuroimaging measures of vascular burden (e.g., white matter hyperintensities) may still introduce residual confounding. Furthermore, although patients were matched for demographic and clinical severity measures, the present cross-sectional design does not allow evaluation of disease progression trajectories or PD biological subtypes. Therefore, it cannot be excluded that PD-DM may represent a subgroup with intrinsically faster progression or more diffuse neurodegeneration due to shared genetic, vascular, metabolic, or environmental mechanisms, rather than a direct effect of diabetes itself. Longitudinal studies integrating multimodal biomarkers and detailed phenotypic characterization will be essential to clarify these relationships.

Limitations notwithstanding, this study has several strengths. First, only newly diagnosed PD patients without any anti-parkinsonian medication were included. Second, two independent cohorts were included to improve results generalizability in both monocentric and multicenter cohorts using different dopaminergic imaging scans. Third, advanced connectivity analyses confirmed and expanded the findings of univariate comparisons, suggesting a mechanism of DM-related connectivity reconfigurations.

Overall, this study suggests that DM may negatively modulate the nigrostriatal system, compromising resilience and accelerating neurodegeneration in PD.

## Supplementary material

Below is the link to the electronic supplementary material.


Supplementary Material 1 (DOCX 52.0 KB)


## Data Availability

The PPMI dataset analysed during the current study are available in the PPMI repository, https://www.ppmi-info.org/. The UniBS dataset generated and/or analysed during the current study are not publicly available due to ethical restrictions but are available from the corresponding author on reasonable request.
