# Supplementary material for: Dopaminergic nigrostriatal vulnerability in Parkinson’s Disease with diabetes: evidence from severity-matched cohorts
Source: Eur J Nucl Med Mol Imaging. 2026 Jun 20;53(11):6274–88. doi: 10.1007/s00259-026-08011-0 (PMC13526104; doi:10.1007/s00259-026-08011-0)
Supplement: Supplementary file 1 [file 259_2026_8011_MOESM1_ESM.docx]

**Supplementary Materials**

**Supplementary Table 1 –** Vascular Risk Factors in the PPMI and UniBS cohorts.

|  | PD-DM | PD-n | Tot. | p-value |
| --- | --- | --- | --- | --- |
| **PPMI cohort (n)** | 56 | 118 | 174 | - |
| Diabetes Mellitus (%) | 56 (100%) | 0 (-) | 56 (32%) | <0.001 |
| Hypertension (%) | 28 (50%) | 27 (23%) | 55 (32%) | <0.001 |
| Hypercholesterolemia (%) | 15 (27%) | 8 (7%) | 23 (13%) | <0.001 |
| Smoking Status (%) | 4 (33%) | 14 (26%) | 18 (10%) | 0.629 |
| Body Mass Index (mean ± SD) | 28.72±5.89 | 26.60±4.47 | 27.29±5.05 | 0.010 |
| **UniBS cohort (n)** | 30 | 65 | 95 |  |
| Diabetes Mellitus (%) | 30 (100%) | 0 (-) | 30 (32%) | <0.001 |
| Hypertension (%) | 20 (66%) | 15 (23%) | 35 (37%) | 0.005 |
| Hypercholesterolemia (%) | 13 (60%) | 18 (27%) | 31 (32%) | 0.007 |
| Smoking Status (%) | 2 (7%) | 8 (12%) | 10 (11%) | 0.121 |
| Body Mass Index (mean ± SD) | 28.07±4.04 | 24.75±3.85 | 26.05±4.23 | <0.001 |

*Abbreviations: PPMI, Parkinson’s Progression Markers Initiative cohort; UniBS, University of Brescia internal cohort.*

**Supplementary Table 2-** Demographic and clinical characteristics of the included patients before matching procedure.

|  | PD-DM | PD-n | p-value |
| --- | --- | --- | --- |
| **PPMI cohort (n)** | 56 | 118 | - |
| Age | 64.73±8.61 | 60.31±10.99 | 0.009 |
| Sex (F/M) | 9/47 | 47/71 | 0.002 |
| DM disease duration^¥^, y | 9.79±8.9 | - | - |
| PD Disease duration, y | 2.40±1.82 | 2.26±2.22 | 0.678 |
| MDS-UPDRS-I | 5.88±4.29 | 4.46±2.91 | 0.011 |
| RBDSQ | 4.77±3.71 | 4.92±2.95 | 0.771 |
| MDS-UPDRS-III | 22.61±2.14 | 20.54±11.16 | 0.191 |
| MoCA | 26.21±2.91 | 27.29±2.14 | 0.035 |
| **UniBS cohort (n)** | 30 | 65 | - |
| Age | 69.88±7.27 | 66.715±6.85 | 0.060 |
| Sex (F/M) | 8/22 | 22/23 | 0.035 |
| DM disease duration*, y | 9.65±6.01 | - | - |
| PD Disease duration, y | 1.52±1.30 | 1.32±1.35 | 0.672 |
| MDS-UPDRS-I | 6.17±4.90 | 4.98±4.41 | 0.280 |
| RBDSQ | 2.71±3.43 | 3.18±3.29 | 0.585 |
| MDS-UPDRS-III | 12.93±9.21 | 13.07±6.59 | 0.942 |
| MoCA | 25.70±2.84 | 26.83±2.02 | 0.039 |

*Continuous variables are expressed as mean ± SD.* ^¥=^ *Available for PD-DM; * =Available only for 20/30 PD-DM patients. p-value was adjusted for age and sex via ANCOVA. Abbreviations: PD, Parkinson’s Disease; DM, Diabetes Mellitus; F, Female; M, Male; MDS-UPDRS, Movement Disorder Society Unified Parkinson’s Disease Rating Scale; RBDSQ= REM Sleep Behaviour Disorder Screening Questionnaire; MoCA = Montreal Cognitive Assessment. PPMI, Parkinson’s Progression Markers Initiative cohort; UniBS, University of Brescia internal cohort.*

**Supplementary Table 3-** Significant voxel-wise differences between PD-DM and PD-n within the two independent cohorts.

|  |  | **MNI coordinates (mm)** | | |  |  |  |
| --- | --- | --- | --- | --- | --- | --- | --- |
| **Cluster size k (mm^3^)** | **Region label** | **x** | **y** | **z** | **t-score** | **Z_(peak)_** | **p-value** |
| **PPMI cohort** |  |  |  |  |  |  |  |
| 289 | Putamen L | -26 | -4 | -4 | 2.40 | 2.40 | 0.009 |
| **UniBS cohort** |  |  |  |  |  |  |  |
| 416 | Caudate R | 18 | 28 | 4 | 3.58 | 3.38 | <0.001 |
|  | Putamen R | 16 | 18 | -8 | 3.02 | 3.02 | <0.001 |
| 261 | Caudate L | -10 | 24 | -4 | 2.89 | 2.78 | 0.003 |
|  | Putamen L | -14 | 24 | 4 | 2.73 | 2.73 | 0.003 |
| 397 | Pallidum L | -18 | -6 | 0 | 2.79 | 2.69 | 0.004 |

*Anatomical labeling was obtained using AAL atlas version 2. Abbreviations: PD, Parkinson’s Disease; DM, Diabetes Mellitus; L, Left; R, Right; PPMI, Parkinson’s Progression Markers Initiative cohort; UniBS, University of Brescia internal cohort.*

**Supplementary Table 4- Dopaminergic binding at baseline by hypertension status**

|  | PD-hypertension | PD-no hypertension | Tot. | p-value |
| --- | --- | --- | --- | --- |
| **PPMI cohort (n)** | 43 | 69 | 112 | - |
| SBR Putamen L | 1.32±0.39 | 1.37±0.48 | 1.35±0.45 | 0.567 |
| SBR Putamen R | 1.24±0.38 | 1.36±0.41 | 1.31±0.39 | 0.159 |
| **UniBS cohort (n)** | 30 | 30 | 60 | - |
| SBR Putamen L | 2.09±0.41 | 1.89±0.44 | 1.97±0.44 | 0.077 |
| SBR Putamen R | 1.84±0.38 | 1.74±0.44 | 1.79±0.41 | 0.635 |

*Continuous variables are expressed as mean ± SD. Abbreviations:L, left; R, right; PD, Parkinson’s Disease; SBR, Specific Binding Ratio; PPMI, Parkinson’s Progression Markers Initiative cohort; UniBS, University of Brescia internal cohort.*

**Supplementary Table 5- Dopaminergic binding at baseline by hypercholesterolemia status**

|  | PD-hypercholesterolemia | PD-no hypercholesterolemia | Tot. | p-value |
| --- | --- | --- | --- | --- |
| **PPMI cohort (n)** | 20 | 92 | 112 | - |
| SBR Putamen L | 1.74±0.61 | 1.54±0.51 | 1.35±0.45 | 0.058 |
| SBR Putamen R | 1.59±0.55 | 1.46±0.43 | 1.31±0.39 | 0.098 |
| **UniBS cohort (n)** | 26 | 34 | 60 | - |
| SBR Putamen L | 1.99±0.47 | 1.97±0.42 | 1.97±0.44 | 0.469 |
| SBR Putamen R | 1.81±0.44 | 1.77±0.39 | 1.79±0.41 | 0.642 |

*Continuous variables are expressed as mean ± SD. Abbreviations:L, left; R, right; PD, Parkinson’s Disease; SBR, Specific Binding Ratio; PPMI, Parkinson’s Progression Markers Initiative cohort; UniBS, University of Brescia internal cohort.*

**Supplementary Table 6- ^123^I-FP-CIT SBR for all ROIs considered as nodes**

|  | CG | PD-DM | PD-n | p-value | Post-hoc |
| --- | --- | --- | --- | --- | --- |
| **PPMI cohort (n)** | 56 | 56 | 56 | - | - |
| SBR Putamen L | 2.25±0.39 | 1.44±0.52 | 1.28±0.35 | 0.024 | *PD-DM≠CG, PD-n ≠CG, PD-DM ≠PD-n* |
| SBR Putamen R | 2.06±0.35 | 1.34±0.46 | 1.28±0.34 | <0.001 | *PD-DM≠CG, PD-n ≠CG* |
| SBR Caudate L | 1.45±0.34 | 0.99±0.41 | 0.96±0.29 | <0.001 | *PD-DM≠CG, PD-n ≠CG* |
| SBR Caudate R | 1.44±0.36 | 0.95±0.37 | 0.95±0.28 | <0.001 | *PD-DM≠CG, PD-n ≠CG* |
| SBR Pallidum L | 3.04±0.55 | 1.99±0.67 | 1.74±0.44 | <0.001 | *PD-DM≠CG, PD-n ≠CG* |
| SBR Pallidum R | 3.13±0.56 | 1.99±0.62 | 1.86±0.49 | <0.001 | *PD-DM≠CG, PD-n ≠CG* |
| SBR Precentral L | 0.34±0.14 | 0.33±0.16 | 0.29±0.12 | 0.188 | - |
| SBR Precentral R | 0.34±0.14 | 0.35±0.19 | 0.31±0.12 | 0.249 | *-* |
| SBR SMA L | 0.32±0.17 | 0.27±0.18 | 0.26±0.13 | 0.103 | *-* |
| SBR SMA R | 0.32±0.19 | 0.28±0.19 | 0.29±0.14 | 0.519 | *-* |
| SBR Thalamus L | 0.66±0.17 | 0.55±0.22 | 0.52±0.17 | <0.001 | *PD-DM≠CG, PD-n ≠CG* |
| SBR Thalamus R | 0.64±0.19 | 0.51±0.19 | 0.49±0.17 | <0.001 | *PD-DM≠CG, PD-n ≠CG* |
| **DNA cohort (n)** | 30 | 30 | 30 | - | - |
| SBR Putamen L | 2.48±0.34 | 2.06±0.38 | 1.88±0.48 | <0.001 | *PD-DM≠CG, PD-n ≠CG, PD-DM ≠PD-n* |
| SBR Putamen R | 2.25±0.29 | 1.85±0.34 | 1.72±0.46 | <0.001 | *PD-DM≠CG, PD-n ≠CG* |
| SBR Caudate L | 1.58±0.34 | 1.55±0.33 | 1.39±0.38 | 0.059 | *-* |
| SBR Caudate R | 1.56±0.29 | 1.49±0.34 | 1.36±0.39 | 0.082 | *-* |
| SBR Pallidum L | 3.34±0.48 | 2.77±0.45 | 2.50±0.64 | <0.001 | *-* |
| SBR Pallidum R | 3.54±0.47 | 2.73±0.56 | 2.54±0.66 | <0.001 | *-* |
| SBR Precentral L | 0.45±0.14 | 0.41±0.21 | 0.37±0.10 | 0.118 | *-* |
| SBR Precentral R | 0.48±0.11 | 0.38±0.21 | 0.37±0.11 | 0.012 | *PD-n ≠CG; PD-DM ≠PD-n* |
| SBR SMA L | 0.38±0.17 | 0.39±0.15 | 0.33±0.22 | 0.385 | *-* |
| SBR SMA R | 0.37±0.15 | 0.43±0.18 | 0.38±0.20 | 0.315 | *-* |
| SBR Thalamus L | 0.83±0.23 | 0.73±0.29 | 0.72±0.16 | 0.167 | *PD-n ≠CG* |
| SBR Thalamus R | 0.85±0.21 | 0.69±0.18 | 0.68±0.30 | 0.132 | *PD-n ≠CG* |

*Continuous variables are expressed as mean ± SD. Post-hoc are Bonferroni corrected for multiple comparisons. Abbreviations: L, left; R, right; SMA, Supplementary Motor Area; PD, Parkinson’s Disease; DM, Diabetes Mellitus; SBR, Specific Binding Ratio; PPMI, Parkinson’s Progression Markers Initiative cohort; UniBS, University of Brescia internal cohort.*
